# Colon cancer cell-derived 12(S)-HETE induces the retraction of cancer-associated fibroblast via MLC2, RHO/ROCK and Ca^2+^ signalling

**DOI:** 10.1007/s00018-016-2441-5

**Published:** 2016-12-24

**Authors:** Serena Stadler, Chi Huu Nguyen, Helga Schachner, Daniela Milovanovic, Silvio Holzner, Stefan Brenner, Julia Eichsteininger, Mira Stadler, Daniel Senfter, Liselotte Krenn, Wolfgang M. Schmidt, Nicole Huttary, Sigurd Krieger, Oskar Koperek, Zsuzsanna Bago-Horvath, Konstantin Alexander Brendel, Brigitte Marian, Oliver de Wever, Robert M. Mader, Benedikt Giessrigl, Walter Jäger, Helmut Dolznig, Georg Krupitza

**Affiliations:** 1 Clinical Institute of Pathology, Medical University of Vienna, Waehringer Guertel 18-20, 1090 Vienna, Austria; 2 Institute of Medical Genetics, Medical University of Vienna, Waehringer Strasse 10, 1090 Vienna, Austria; 3 Department for Clinical Pharmacy and Diagnostics, Faculty of Life Sciences, University of Vienna, Althanstrasse 14, 1090 Vienna, Austria; 4 Department of Medicine I, Comprehensive Cancer Centre, Medical University of Vienna, Waehringer Guertel 18-20, 1090 Vienna, Austria; 5 Department of Pharmacognosy, Faculty of Life Sciences, University of Vienna, Althanstrasse 14, 1090 Vienna, Austria; 6 Neuromuscular Research Department, Centre of Anatomy and Cell Biology, Medical University of Vienna, Waehringer Strasse 13, 1090 Vienna, Austria; 7 Department of Medicine I, Institute of Cancer Research and Comprehensive Cancer Centre, Medical University of Vienna, Borschkegasse 8a, 1090 Vienna, Austria; 8 Department of Radiation Oncology and Experimental Cancer Research, Ghent University, De Pintelaan 185, 9000 Ghent, Belgium

**Keywords:** 3D invasion model, Tumour progression, ECM, Arachidonic acid metabolite, Signal transduction

## Abstract

Retraction of mesenchymal stromal cells supports the invasion of colorectal cancer cells (CRC) into the adjacent compartment. CRC-secreted 12(S)-HETE enhances the retraction of cancer-associated fibroblasts (CAFs) and therefore, 12(S)-HETE may enforce invasivity of CRC. Understanding the mechanisms of metastatic CRC is crucial for successful intervention. Therefore, we studied pro-invasive contributions of stromal cells in physiologically relevant three-dimensional in vitro assays consisting of CRC spheroids, CAFs, extracellular matrix and endothelial cells, as well as in reductionist models. In order to elucidate how CAFs support CRC invasion, tumour spheroid-induced CAF retraction and free intracellular Ca^2+^ levels were measured and pharmacological- or siRNA-based inhibition of selected signalling cascades was performed. CRC spheroids caused the retraction of CAFs, generating entry gates in the adjacent surrogate stroma. The responsible trigger factor 12(S)-HETE provoked a signal, which was transduced by PLC, IP3, free intracellular Ca^2+^, Ca^2+^-calmodulin-kinase-II, RHO/ROCK and MYLK which led to the activation of myosin light chain 2, and subsequent CAF mobility. RHO activity was observed downstream as well as upstream of Ca^2+^ release. Thus, Ca^2+^ signalling served as central signal amplifier. Treatment with the FDA-approved drugs carbamazepine, cinnarizine, nifedipine and bepridil HCl, which reportedly interfere with cellular calcium availability, inhibited CAF-retraction. The elucidation of signalling pathways and identification of approved inhibitory drugs warrant development of intervention strategies targeting tumour–stroma interaction.

**Electronic supplementary material:**

The online version of this article (doi:10.1007/s00018-016-2441-5) contains supplementary material, which is available to authorized users.

## Introduction

The risk to develop colorectal cancer (CRC) is high and the danger to die from metastases is evident. The multistage nature of CRC development [1, 2] defines a number of tumour suppressor genes and (proto)-oncogenes that become de-regulated (i.e. by mutations, epigenetic modulation of gene expression, or other), and once gene expression changes in a certain spatio-temporal order the cancer progresses stepwise in the well-known sequence: aberrant crypt foci—adenoma—primary cancer—metastasising carcinoma with the tumour leaving the hitherto defined area. The vast mechanistic complexity of metastatic spreading involves the dissolution of the basement membrane, invasion into the surrounding stroma, intravasation into blood and lymphatic vessels, circulation and survival in these vessels, extravasation and eventually colonialisation of a distant secondary site. The tumour–stroma is critically involved in these processes and there is compelling evidence for a cancer-associated fibroblast (CAF) gene signature as adverse prognostic marker in CRC [3, 4]. Thus, CRC needs the cooperation of stromal cells to facilitate metastatic dissemination, yet the mechanisms are poorly understood. A suspect pro-metastatic factor, which is secreted by tumour cells and that takes on control over stromal endothelial cells, is 12(S)-HETE. This pro-inflammatory arachidonic acid metabolite perturbs inter-cellular junctions [5] and causes the retraction of lymphatic and blood endothelial cell walls. Therefore, 12(S)-HETE is called “endothelial retraction factor” [6] and may facilitate CRC intravasation and lymph node metastasis [7]. Further, 12(S)-HETE, which is metabolised by the lipoxygenases ALOX12 and ALOX15 [8] and by cytochrome-P450-1A1 (CYP1A1) [9, 10], induces the metastatic spreading of prostate carcinoma cells [11]. 12(S)-HETE levels are not significantly different in the tissue specimen of normal, polyp and cancer mucosa [12], which is in accordance with similar ALOX12 expression in normal glandular colon cells and CRC tissue as detected by immunohistochemistry (http://www.proteinatlas.org). However, the CRC cell lines Caco2, SW480 and SW620 express steadily increasing levels of ALOX12 and secrete increasing amounts of 12(S)-HETE, respectively, and this is directly proportional to their metastasising potential i.e. increased growth in soft agar and enhanced migration [13]. In line with this observation, CYP1A1 mRNA is over-expressed in CRC liver metastases as compared to normal colonic epithelium, as detected by RNASeq, whereas CYP1A1 mRNA and protein expression does not vary, between normal colonic tissue and CRC. Hence, there may exist a direct correlation between 12(S)-HETE production and metastatic spreading. This is further supported by the finding that 12(S)-HETE activates the pro-metastatic transcription factor ZEB1 in endothelial cells, assisting the formation of cancer spheroid-triggered “circular chemorepellent induced defects” (CCIDs) [14], which resemble entry gates for the tumour into the vasculature [15]. Conversely, the inhibition of ZEB family expression by miR200 in endothelial cells reduces their potential to form CCIDs [7]. Taken together there is ample evidence that 12(S)-HETE and the ZEB pathway contribute to malignancy and therefore, studying the interface between cancer cells and their immediate stromal environment is relevant to understand tumour spreading.

In the lymph node metastases of breast cancer patients, the production of 12(S)-HETE correlates directly with increased ALOX15 expression and inversely with metastasis-free survival. Moreover, high ALOX12 expression in the lymph node metastases of breast cancer patients inversely correlates with metastasis-free survival as well [15]. The contribution of ALOX15 to lymph node metastases was furthermore proven in SCID mice, which were orthotopically transgrafted with MCF-7 cells in which ALOX15 [the major 12(S)-HETE producing enzyme in this cell line] was knocked-down [15].

Physiologically, neutrophils and activated macrophages secrete 12(S)-HETE, which causes endothelial barrier retraction and enables their transmigration through the vasculature to reach sites of inflammation [5, 16]. Apparently, cancer cells can co-opt this property as cancer cell-secreted 12(S)-HETE activates MLC2 in lymph endothelial cells as a prerequisite for endothelial junction retraction and formation of CCIDs [5, 17–19].

Here, we highlighted the role of 12(S)-HETE and the response of CAFs in this inter-cellular communication and tried to adapt a 3D intravasation model, to study mechanisms of 12(S)-HETE-triggered CRC spheroid invasion into the CAF compartment. We demonstrate that CAFs retract from CRC cell spheroids and form CCIDs and identified signalling pathways that steer this process.

## Materials and methods

### Antibodies and reagents

Polyclonal rabbit anti-phospho-myosin light chain 2 (Ser19) and polyclonal rabbit anti-myosin light chain 2 were purchased from Cell Signaling (Danvers, MA, USA), monoclonal mouse anti-MYLK was from Santa Cruz Biotechnology (Heidelberg, Germany), polyclonal rabbit anti-BLT2 antibody was from Sigma (Munich, Germany), polyclonal rabbit anti-12(L)-HETE antibody (L-configuration is identical to S-configuration for 12-HETE) from Enzo Life Sciences (Lausen, Switzerland), monoclonal mouse anti-cytokeratin-20 (CK20) antibody from Neomarker (Thermo Fisher Scientific, Inc., Waltham, MA, USA), monoclonal mouse anti-tenascin C antibody from Novocastra (Thermo Fisher Scientific, Inc., Waltham, MA, USA), and polyclonal rabbit anti-GAPDH was from Trevigen (Gaithersburg, MD, USA). Polyclonal swine anti-rabbit IgG was purchased from Dako (Glostrup, Denmark) and polyclonal Alexa 488 goat anti-mouse and Alexa 594 goat anti-rabbit antibodies were purchased from Molecular Probes (Thermo Fisher Scientific, Inc., Waltham, MA, USA).

siRNAs targeting MLC2 (MYL2; SMART pool, ON-TARGET PLUS, Cat. No.: L-011087000005) and MYLK (SMART pool, ON-TARGET PLUS, Cat. No.: L-0053510000) were ordered from Dharmacon (Gene Expression and Gene Editing, GE Healthcare, Lafayette, CO, USA), and non-targeting control siRNA (Silencer Select Negative Control No. 1 siRNA, Cat. No.: 4390843) was from Ambion (Life Technologies, Carlsbad, CA, USA).

Baicalein, BAPTA-AM, edelfosine, KN-62 and Y27632 were purchased from Sigma Aldrich (Munich, Germany), blebbistatin, U73122 and rhosin were from Calbiochem (Merck Millipore, Darmstadt, Germany), bepridil hydrochloride (B5016), cinnarizine (C5270), carbamazepine (C8981) and nifedipine (N7634) from Sigma Aldrich (Munich, Germany). 12(S)-HETE was purchased from Cayman Chemical (Ann Arbor, MI, USA), and cell-tracker Green CMFDA Dye and cell-tracker Red CMTPX Dye were ordered from Invitrogen (Karlsruhe, Germany).

### Cell culture

Human SW620 (CCL-227, lymph node metastasis) and SW480 (CCL-228, primary tumour) colorectal adenocarcinoma cells were obtained from American Type Culture Collection (ATCC, Rockville, MD, USA) and grown in RPMI or DMEM, respectively, supplemented with 10% foetal calf serum (FCS), 1% penicillin/streptomycin (PS), and l-glutamine. Human colon adenoma cells, LT-97 [20], were cultivated in WNRE medium [RPMI/10% FCS supplemented with conditioned medium of L cells producing recombinant WNT3A, Noggin and R-spondin (10% each) supplemented with human recombinant EGF (50 ng/ml, PeproRech)]. Human Lung Fibroblasts (HLFs, CCL-135) were purchased from American Type Culture Collection (ATCC, Rockville, MD, USA) and maintained in DMEM supplemented with 10% FCS, 1% PS and 1% l-glutamine. Human primary CAF (CT5.3) were isolated from a colorectal adenocarcinoma resection specimen obtained in accordance with the local ethics committee (Ghent University Hospital). In short, tissue fragments were cut in small pieces and transferred into a six-well plate with FCS. Isolated CT5.3 were infected with a pBABE retroviral vector expressing the hTERT open reading frame [21].

CT5.3 cells were cultivated in DMEM supplemented with 10% FCS, 1% PS, and 1% l-glutamine and 2 µg/ml puromycin. Primary CAFs (CAF3) [22] and human dermal microvascular endothelial cells (BECs; C-12260, Promocell, Heidelberg, Germany) were maintained in EGM2 MV (Clonetics CC-4147, Allendale, NJ, USA). All cells were kept at 37 °C in a humidified atmosphere containing 5% CO_2_.

### Spheroid formation

SW480 cells (1500 cells per spheroid) or SW620 cells (2000 cells per spheroid) were transferred into DMEM (supplemented with 5% FCS, 1% PS and 1% l-glutamine) containing methylcellulose (M-512; Sigma Aldrich) at a final concentration of 0.3%. 150 µl of this cell suspension was transferred to each well of a 96-well round bottom plate (Greiner Bio-one, Cellstar 650185, Kremsmünster, Austria). The plates were centrifuged for 30 min at 1000 rpm and thereafter incubated for 4 days to allow spheroid formation.

LT-97 cells (2000 cells per spheroid) were transferred into WNRE medium without methylcellulose and 150 µl of this cell suspension seeded into each well of a 96-well, non-adhesive, round bottom plate (Nunclon Sphera ULA plates). The plates were centrifuged for 30 min at 1000 rpm and thereafter incubated for 4 days to allow spheroid formation.

### Colon cancer invasion model (collagen gel culture)

Nylon meshes were prepared from nylon mesh inserts of Medicon syringe filters (100 µm, Becton–Dickinson) of which a hole of 1 cm diameter was cut out in the centre. The nylon meshes were autoclaved and placed onto the inside surface of lids of 24-well plates. CT5.3 cells were stained with cell-tracker green (Invitrogen, Karlsruhe, Germany), trypsinised and counted. A total of 4 × 10^5^ cells for each gel were transferred into 1.5-ml Eppendorf tubes and centrifuged at 1000 rpm for 5 min at 4 °C. For collagen gel preparation, all steps were performed on ice. Collagen solutions were prepared by mixing 2 mg/ml collagen I (Corning, collagen I, rat tail, Bedford, MA, USA), 10% 10× PBS and DMEM and were neutralised by the addition of 7 µl/ml 1 M NaOH. A total of 300 µl each of the collagen solution was transferred into the tubes containing the cell pellets which were gently re-suspended. The collagen/cell suspensions were carefully transferred on the nylon meshes and incubated for 30 min at 37 °C/5% CO_2_ to allow the collagen solutions to solidify. Thereafter, 1 ml DMEM was added into each well and gels were incubated for 24 h. Then, BECs were stained with cell-tracker red, trypsinised and counted. 2 × 10^5^ cells were transferred on top of each collagen gel and the co-cultures were incubated for another 24 h. SW620 spheroids were prepared as described above, washed and carefully placed on top of the BEC monolayer. 24 h after spheroid transfer, CCID areas in BECs and CAFs were analysed using a fluorescence microscope.

### Circular chemo-repellent-induced defect (CCID) assay

In this assay, the sizes of cell free areas (circular chemo-repellent induced defects; CCIDs) which are formed in the CAF monolayer directly underneath the tumour spheroids were measured. Primary CAFs, CT5.3 cells or HLFs were grown to confluence in 24-well plates and subsequently stained with cell-tracker green. Colon cancer spheroids were washed with PBS and transferred on top of the CAF or HLF monolayers. After 6 h of co-cultivation, CCID areas were photographed using an Axiovert (Zeiss, Jena, Germany) fluorescence microscope to visualise the cell-tracker stained CAFs or HLFs. CCID areas were calculated using Zen Little 2012 software (Zeiss, Jena, Germany). For each condition, the CCID areas of at least 15 spheroids were measured.

### Transfection of primary CAFs and CT5.3 cells

Cells were seeded in 24-well plates and grown to 70% confluence. A total of 0.75 µg siRNA and 4 µl HiPerFect Transfection Reagent (QIAGEN, Cat. No.: 301705) were mixed in 100 µl serum-free medium and incubated for 30 min at room temperature to allow the formation of transfection complexes, which were then carefully added onto the cells (to a final siRNA concentration of 100 nM). The cells were incubated for 48 h at normal growth conditions and were subsequently used for CCID assays or RNA isolation.

### Quantitative RT-PCR (qPCR)

Cells were harvested after transfection, and RNA was isolated using the RNeasy Mini Kit 50 and QIAshredder 50 (QUIAGEN, Hamburg, Germany). The final RNA concentration was measured using a NanoDrop Fluorospectrometer (Thermo Fisher Scientific, Inc., Waltham, MA, USA). An amount of 2 µg of total RNA was reverse transcribed using RNA to cDNA EcoDry Premix Protocol-At-A-Glance (Clontech, 2 Saint-Germain-en-Laye, France). The resulting cDNA was amplified using TaqMan Gene Expression Master Mix (Applied Biosystems, Vienna, Austria). The PCR products were analysed on the Chromo4 PCR System (Bio-Rad, Hercules, CA, USA). The following TagMan probes were used: GAPDH (Hs99999905_m1) and MYLK (Hs00364926_ma). qPCR was performed in triplicate for each cDNA template. Gene expression was normalised to GAPDH expression (glyceraldehyde 3-phosphate dehydrogenase) and was calculated using the ∆∆*C*_T_ method.

### SDS gel electrophoresis and Western blotting

CT5.3 cells were grown in T-25 tissue culture flasks (Nunc, Roskilde, Denmark) or six-well plates to 80% confluence and subsequently starved for 24 h. Then, cells were pre-treated with respective inhibitors or solvent for 1 h and stimulated with 1 µM 12(S)-HETE for another 15 min. Afterwards, cells were washed twice with ice-cold PBS and lysed in buffer containing 50 mM Tris–HCl (pH 6.8), 6% SDS, 20% glycerine, 1.85 mM EDTA, phosphatase inhibitor cocktail, and protease inhibitor cocktail. For complete cell lysis, the mixture was sonicated 5–10 times on ice. The lysate was stored at −20 °C until further analysis. Equal amounts of protein were separated by SDS polyacrylamide gel electrophoresis and electro-transferred onto Amersham Hybond-P PVDF transfer membrane (GE Healthcare, Freiburg, Germany) at 100 V for 1 h in ice-cold transfer buffer [containing 20 mM Tris-base, 150 mM glycine, 20% (v/v) methanol, pH 8.5]. Membranes were stained with Ponceau S (Sigma-Aldrich, Munich, Germany) to control transfer efficiency and equal sample loading. After washing with TBS-T (Tris Buffered Saline/Tween 20; pH 7.6), membranes were immersed in blocking solution (5% non-fat dry milk in TBS containing 0.1% Tween20) at room temperature for 1 h. Then, membranes were washed and incubated with primary antibodies (5% BSA in TBS-T; 1:500–1:1000) by gently rocking at 4 °C overnight. Thereafter, membranes were washed and incubated with secondary antibodies (peroxidase-conjugated swine anti-rabbit IgG, dilution 1:5000) at room temperature for 1 h. Chemo-luminescence was developed by Amersham ECL prime Kit (GE Healthcare, Freiburg, Germany) and detected using a Lumi-Imager F1 Workstation (Roche, Basel, Switzerland). Densitometry of Western blots was analysed with Image-J software (National Institutes of Health, Maryland, USA).

### Immunofluorescence

Fresh unfixed human tissue was stored in nitrogen and sectioned in 3-µm-thick slices using a Cryostar NX50 (Thermo Fisher Scientific, Inc., Waltham, MA, USA). Sections were then transferred on Superfrost-Plus object slides (Thermo Fisher Scientific, Inc., Waltham, MA, USA) and dried for 10 min at room temperature (RT). Then samples were incubated with primary antibodies (1:50 in Tris buffer pH 7.2) for 30 min at RT. After washing three times with Tris buffer, samples were incubated with secondary antibodies for another 30 min at RT. Then, slides were washed again and incubated with DAPI (1:50,000) for 1 min. Thereafter, samples were embedded with GelTol, coverslipped and analysed on a Zeiss LSM5-Exiter confocal microscope (Zeiss, Jena, Germany). Surgical specimens were collected from three patients who underwent surgery for colorectal cancer and gave their informed consent. The study protocol was approved by the ethics committee of the Medical University of Vienna (EK 1659/2012).

### Intracellular Ca^2+^ assay

Free intracellular Ca^2+^ levels were measured using FluoForte Calcium Assay Kit (Enzo Life Science, Ann Abor, MI, USA). 3 × 10^4^ CT5.3 cells/well/100 µl DMEM were seeded into 96-well black-wall clear-bottom plates (Nunc, Thermo Scientific, NY, USA). After 24 h, cells were pre-treated with respective inhibitors or solvent for 1 h and subsequently incubated in 100 µl FluoForte Dye for 45 min at 37 °C and 15 min at room temperature. Then, cells were stimulated with 1 µM 12(S)-HETE and fluorescence was measured at 490/525 nm using a fluorescence plate reader.

### 12(S)-HETE assay

DLD-1 and SW620 cells were seed in six-well plates and grown in 2.5 ml complete MEM medium to ~70–80 confluence when FCS was removed overnight. Then, cells were treated with 10 µM arachidonic acid (Sigma-Aldrich, Munich, Germany) in serum-free medium for another 4 h. The concentration of 12(S)-HETE in the cellular supernatant was measured with minor modifications as described previously [23] using the 12(S)-HETE enzyme immunoassay kit (Enzo Life Sciences, Lausen, Switzerland). Absorbance was measured with a Wallac 1420 Victor 2 multilabel plate reader (Perkin Elmer Life and Analytical Sciences, Shelton, CT, USA). The concentration of 12(S)-HETE in the cellular supernatant was normalsed to cell number to account for differences in the cell number.

### Statistical analysis

For statistical analyses, Excel 2013 software and Prism 6 software package (GraphPad, San Diego, CA, USA) were used. The values were expressed as mean ± SEM and one-way ANOVA and student’s *t* test was applied to compare differences between control samples and treatment groups. Statistical significance level was set to *p* < 0.05.

## Results

### Establishment of a 3D in vitro model resembling CRC invasion into stroma

**Fig. 1:**
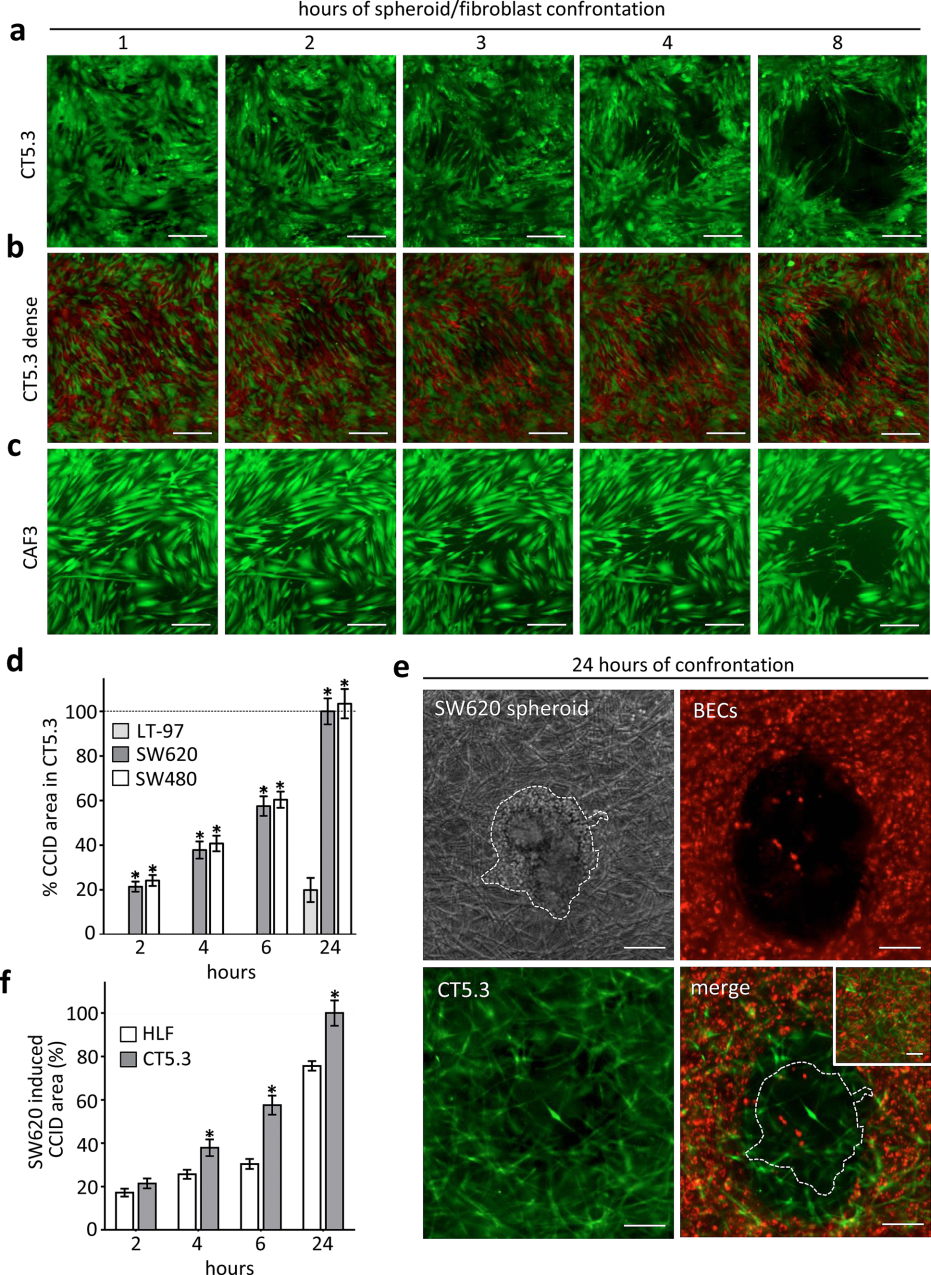
Colon cancer spheroid-induced CCID formation in CAFs SW620 spheroids was transferred on **a** cell-tracker (*green*) stained CT5.3 or **b** more densely seeded CT5.3 (50% of CT5.3 were stained with *green* cell-tracker the other 50% were stained with *red* cell-tracker) or **c** on cell-tracker (*green*) stained primary CAF3 layers. The formation of CCIDs was monitored at different time points by fluorescence microscopy. The formation of CCIDs was monitored at different time points by fluorescence microscopy. Representative pictures of at least three independent experiments are shown. **d** Quantification of SW620, SW480 and LT-97 induced CCID formation in CT5.3 monolayers. **e** Collagen gel culture of CT5.3 cells, BECs and SW620 spheroids. Cell-tracker (*green*) stained CT5.3 cells were embedded in collagen I gel. Then, cell-tracker (*red*) stained BECs were seeded on top of these gels. Subsequently, SW620 spheroids were placed on top and the formation of CCIDs was monitored after 24 h by fluorescence microscopy. Cultures without SW620 spheroids were used as controls (*small inset*). **f** Quantification of SW620 spheroid-induced CCID formation in CT5.3 and HLF monolayers. *Scale bars* 200 µm. *Bar graphs* represent means, *error bars* indicate ± SEM, *asterisks* significance compared to control (*p* < 0.05; *t* test or ANOVA)

It was shown that cancer cells repel endothelial cells [15], and here we investigated whether fibroblasts may as well retract from CRC spheroids. SW620 spheroids were placed on top of immortalised CT5.3 CAFs [21], which induced their retraction within 6 h thereby forming CCIDs (Fig. 1a). In patients, the stroma around CRC mucosa often consists of densely packed fibroblasts. Therefore, CT5.3 CAFs were cultivated more densely and also under these conditions CAFs were forced by SW620 spheroids to retract, although the CCID areas were smaller than in less densely grown CAFs (Fig. 1b; in order to better distinguish individual fibroblasts in this setting 50% of CT5.3 were stained with cell-tracker red and 50% with cell-tracker green). Also, primary CAFs (CAF3) [22] responded with retraction when SW620 spheroids were placed on top (Fig. 1c). Furthermore, SW480 spheroids (SW480 is the primary tumour of the matching SW620 lymph node metastasis)-induced CCIDs in CT5.3 cell layers, which demonstrates the potential also of the primary tumour to invade the surrounding stroma (Fig. 1d). In contrary, non-cancerous LT-97 adenoma cells [20] did not trigger CCID formation in the CAF barrier within 6 h (Fig. 1d). To stress the model further, CAFs were covered with collagen I, which mimics the stroma/matrix histopathology surrounding CRCs. Then, SW620 spheroids were placed on top, which still caused the retraction of CT5.3 cells thereby establishing a cell-free area (not shown). In addition, blood endothelial cells (BECs) were included to this model and seeded onto the surface of collagen I-embedded CAFs. On top of this co-culture system, SW620 spheroids were placed. Under these conditions, the spheroids induced the formation of CCIDs in BECs and also the retraction of the subjacent fibroblasts (Fig. 1e). The setting of this in vitro model closely resembles the patho-physiology in humans when CRC cells extravasate the blood vasculature and invade a distant organ. Metastatic CRC especially invades liver and lungs. Indeed, SW620 spheroids caused also the retraction of normal human lung fibroblasts (HLFs) even if these cells were less responsive when compared with CAFs (Fig. 1f). Therefore, the phenomenon of CCID formation is not restricted to colon CAFs as also EC junction retraction was due to CRC-secreted 12(S)-HETE [7]. To localise 12(S)-HETE in human normal and cancerous colonic epithelia, fresh cryo-preserved and non-fixed tumour tissues were analysed by immunofluorescence and laser scanning microscopy. In normal as well as in cancerous colonic mucosa cells, 12(S)-HETE was detected (Supplementary Fig. S1). A quantification by this method was not possible, as the harsh conditions of the colon environment certainly degraded most of the 12(S)-HETE molecule (which is rapidly oxidised at four different double bonds etc.), but in general, these data are in agreement with HPLC data, which demonstrate that normal colon-, polyp- and colon cancer mucosa produce similar amounts of 12(S)-HETE [12]. The level of the 12(S)-HETE concentration in metastases of CRC remains unexplored, since metastases are not surgically removed in the clinical routine. To this end, it is of particular interest that RNA sequencing revealed CYP1A1 mRNA over-expression in CRC liver metastases as compared to normal colonic epithelium (http://www.ebi.ac.uk/gxa/experiments/E-GEOD-50760?geneQuery=ENSG00000140465&queryFactorValues=g2_g1&_specific=on).

Given that CCID formation was demonstrated in all variations of our experiments and that 12(S)-HETE was detected in CRC tissue, the model was reduced in the next step (as shown in Fig. 1a, c) to investigate the underlying mechanism causing retraction.

### 12(S)-HETE-activated MLC2 triggers CCID formation in the CRC-stroma invasion model

**Fig. 2:**
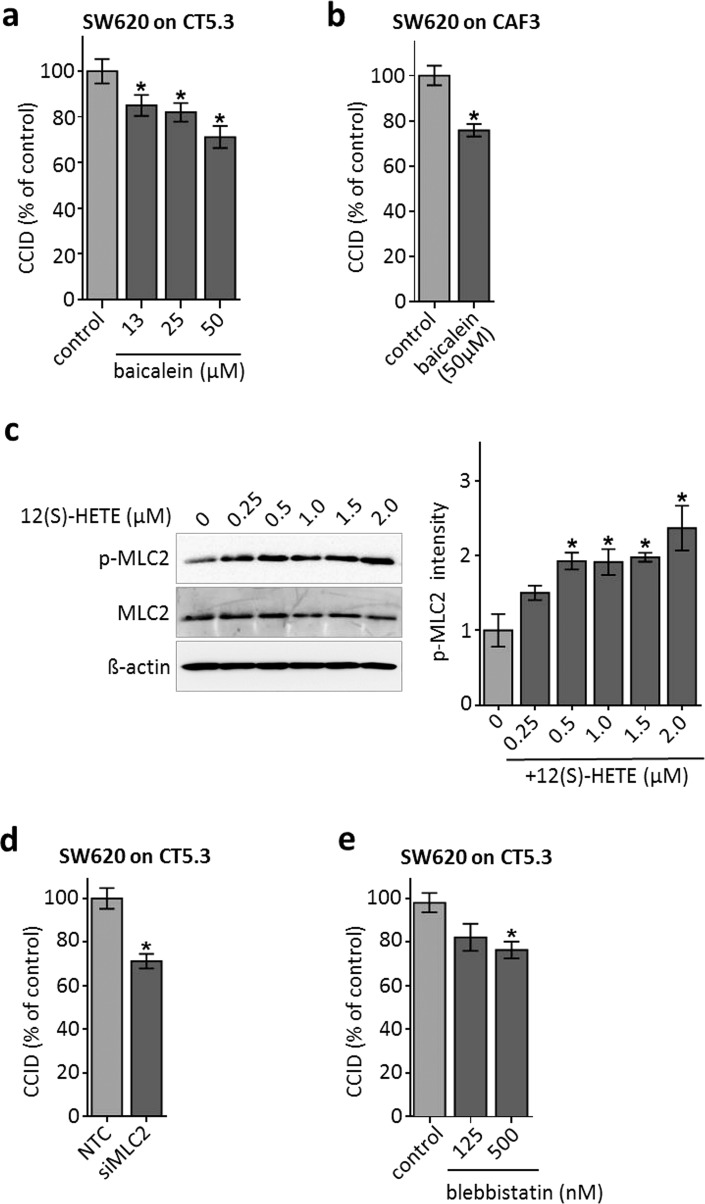
CCID formation in CT5.3 and CAF3 is inhibited by baicalein and depends on MLC2. SW620 spheroids were pre-treated with baicalein at indicated concentrations or solvent (control; DMSO) and transferred on cell-tracker stained **a** CT5.3 or **b** CAF3 monolayers. After 6 h CCID areas were measured. **c** CT5.3 cells were stimulated with 0.25, 0.5, 1.0, 1.5 and 2.0 µM 12(S)-HETE or solvent (0) for 20 min. Western blotting was used to determine MLC2 phosphorylation at serine 19. Equal sample loading was controlled by MLC2 total protein and GAPDH. Phospho-MLC2 (p-MLC2) was quantified by densitometry and normalised to MLC2 and GAPDH. Solvent treated control was set to 1. **d** CT5.3 cells were transfected with either non-targeting RNA (NTC) or siRNA targeting MLC2 (siMLC2). After 24 h SW620 spheroids were transferred on top of the CT5.3 monolayers and after 6 h co-cultivation CCID areas were measured. **e** CT5.3 cells were pre-treated with blebbistatin at indicated concentrations or solvent control (control; DMSO). Then, SW620 spheroids were placed on top of the CT5.3 cell monolayers for 6 h and CCID areas were measured. *Bar graphs* represent means, *error bars* indicate ± SEM, *asterisks* significance compared to control (*p* < 0.05; *t* test)

Metastatic SW620 cells were shown to express ALOX12 and to secrete 12(S)-HETE. SW480 and the well-differentiated CaCo2 cells (both derived from primary tumour sites) express less ALOX12 and produce only half the amount of 12(S)-HETE as compared to SW620 cells [13]. Also, DLD-1 cells (derived from a primary tumour site) secreted lower levels of 12(S)-HETE [7.3 ng/ml (23 nM)] than metastatic SW620 cells [10.6 ng/ml (33 nM)] within 4 h (1 × 10^6^ cells, each). This suggests a direct correlation between higher 12(S)-HETE production and increasing malignancy. However, this did not correlate with their CCID-forming potential, as SW60 and SW480 spheroids induced CCID formation alike (Fig. 1d) and this implicated that both cell types may have produced an overload of 12(S)-HETE, which triggered maximal fibroblasts retraction. In the immediate proximity of CAFs, the concentration of 12(S)-HETE that was secreted by SW620 spheroids must have been much higher than 33 nM at least in the in vitro setting studied here. To confirm the contribution of 12(S)-HETE upon SW620 spheroid-triggered CCID formation within the CAF barrier, ALOX12, a major producer of 12(S)-HETE, was inhibited by baicalein. In the CRC/CAF invasion model using immortalised CT5.3 fibroblasts as well as primary CAF3, baicalein attenuated the formation of CCIDs (Fig. 2a, b). Therefore, ALOX12 in SW620 cells, and consequently 12(S)-HETE, induced CCID formation in CAF barriers similar to that induced in EC barriers [7]. EC retraction and CCID formation depend on the expression and activity of the mobility marker myosin light chain 2 (MLC2) [24] and we hypothesised that this might also be the case in CAFs. Indeed, the treatment of CT5.3 cells with 0.25–2.0 µM (80–638 ng/ml) 12(S)-HETE triggered the phosphorylation of MLC2 at serine 19, indicating its activation (Fig. 2c). Therefore, CAFs were further on treated with a standardised concentration of 1 µM 12(S)-HETE to study the mechanisms of their retraction and CCID formation. MLC2 was essential for CAF retraction, since siRNA-mediated knock-down of MLC2 expression (siMLC2) reduced the CCID areas (Fig. 2d; proper knock-down of MLC2 is shown in supplementary Fig. S2). Inhibition of MLC2 activity by blebbistatin (Fig. 2e) significantly inhibited CCID formation in the CAF barrier as well, which further substantiated the contribution of MLC2 to CAF retraction.

### 12(S)-HETE-triggered Ca^2+^ release induces MLC2 activation and CCID formation

**Fig. 3:**
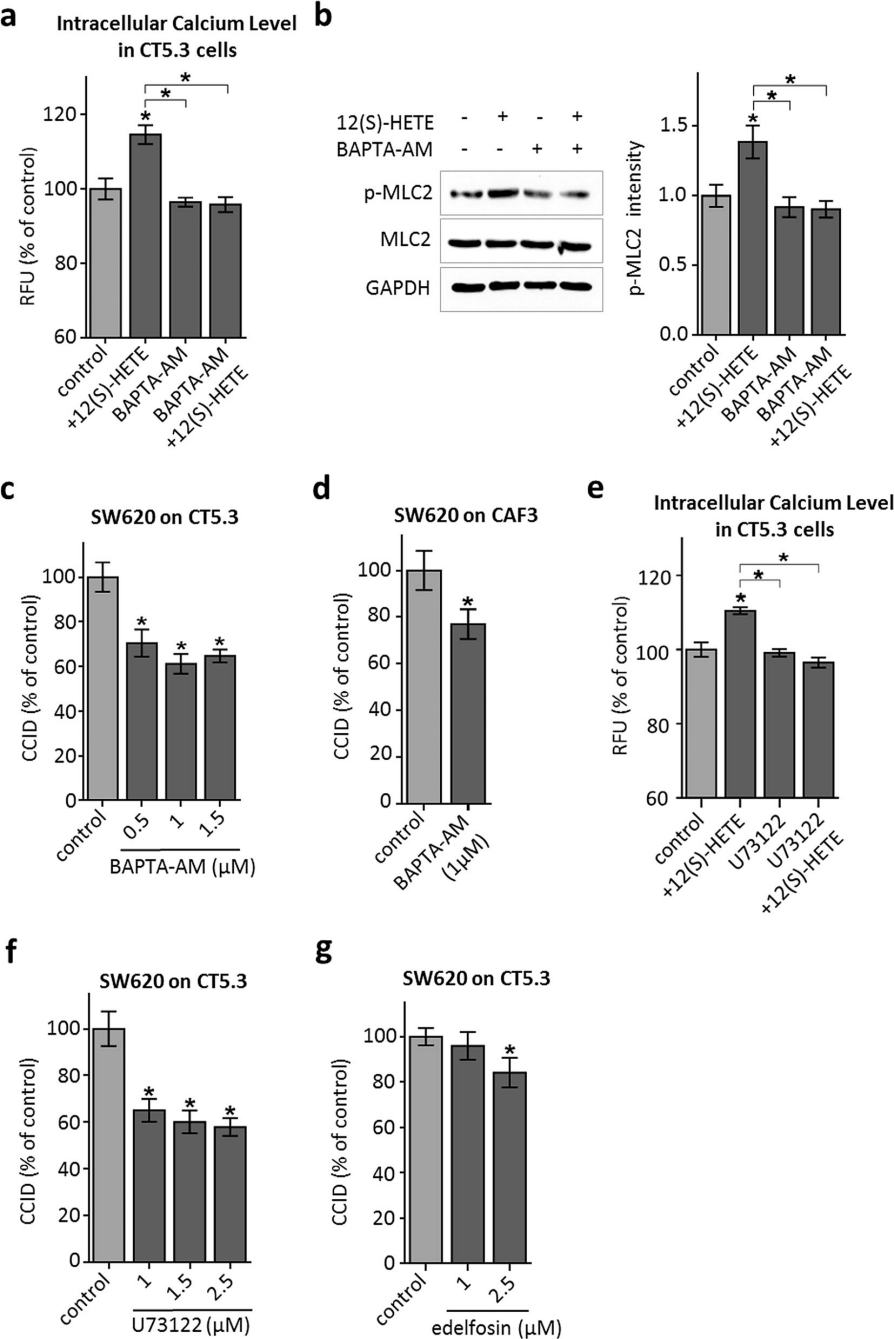
Analysis of the 12(S)-HETE-induced Ca^2+^ pathway in CT5.3 cells. **a** CT5.3 were pre-treated with 2.5 µM BAPTA-AM, 2.5 µM U73122 or DMSO and subsequently incubated with FluoForte™ Dye. Then, cells were stimulated with 1 µM 12(S)-HETE or solvent (control; DMSO) and intracellular-free calcium was measured after 3 min. **b** CT5.3 were pre-treated with 2.5 µM BAPTA-AM or DMSO and stimulated with 1 µM 12(S)-HETE or solvent (control; DMSO). After 15-min cells were lysed, proteins separated by SDS gel electrophoresis and analysed by Western blotting was used to determine MLC2 phosphorylation at serine 19. Equal sample loading was controlled by MLC2 total protein and GAPDH. Phospho-MLC2 (p-MLC2) was quantified by densitometry and normalised to MLC2 and GAPDH. Solvent-treated control was set to 1. **c**–**f** CAFs were pre-treated with the intracellular Ca^2+^ chelator BAPTA-AM, U73122 (inhibiting the generation of IP3) and the PLC inhibitor edelfosine at indicated concentrations or DMSO (control). Then, SW620 spheroids were placed on top of the CAF monolayers for 6 h and CCID areas were measured. *Bar graphs* represent means, *error bars* indicate ± SEM, *asterisks* significance compared to control (*p* < 0.05; *t* test or ANOVA)

12(S)-HETE induces Ca^2+^ release in normal human fibroblasts [25], HEK239 cells [26] and in CHO cells through the BLT2 low affinity receptor [27], and was reported to activate L-type calcium channels in renal myocytes [28]. BLT2 is expressed in the colon cancer-associated fibroblasts, CT5.3, as well (Supplementary Fig. S3) and accordingly, Ca^2+^ levels increased significantly in 12(S)-HETE-treated CT5.3 cells (Fig. 3a). The co-treatment with the specific intracellular Ca^2+^ chelator BAPTA-AM evidenced that Ca^2+^ signalling was responsible for 12(S)-HETE-induced Ser19-MLC2 phosphorylation (Fig. 3b) and furthermore, BAPTA-AM inhibited CRC-induced CCID formation in CT5.3 (Fig. 3c) and CAF3 (Fig. 3d). The immediate upstream factor triggering Ca^2+^ release from intracellular stores is inositol-3-phosphate (IP3) and the IP3-generating enzyme phospholipase-C-beta (PLCβ). Consistently, the inhibition of IP3 generation by U73122 significantly attenuated Ca^2+^ release (Fig. 3e) and CRC-triggered CCID formation (Fig. 3f) and the PLC inhibitor edelfosine attenuated the retraction of CT5.3 cells as well (Fig. 3g). In addition, both inhibitors, U73122 and edelfosine, inhibited the phosphorylation of MLC2 (Supplementary Fig. S4). Therefore, the PLCβ–IP3–Ca^2+^–MLC2 pathway is critically involved in 12(S)-HETE-induced CCID formation in CAFs.

### ROCK transduces 12(S)-HETE-triggered MLC2 activation and CCID formation

**Fig. 4:**
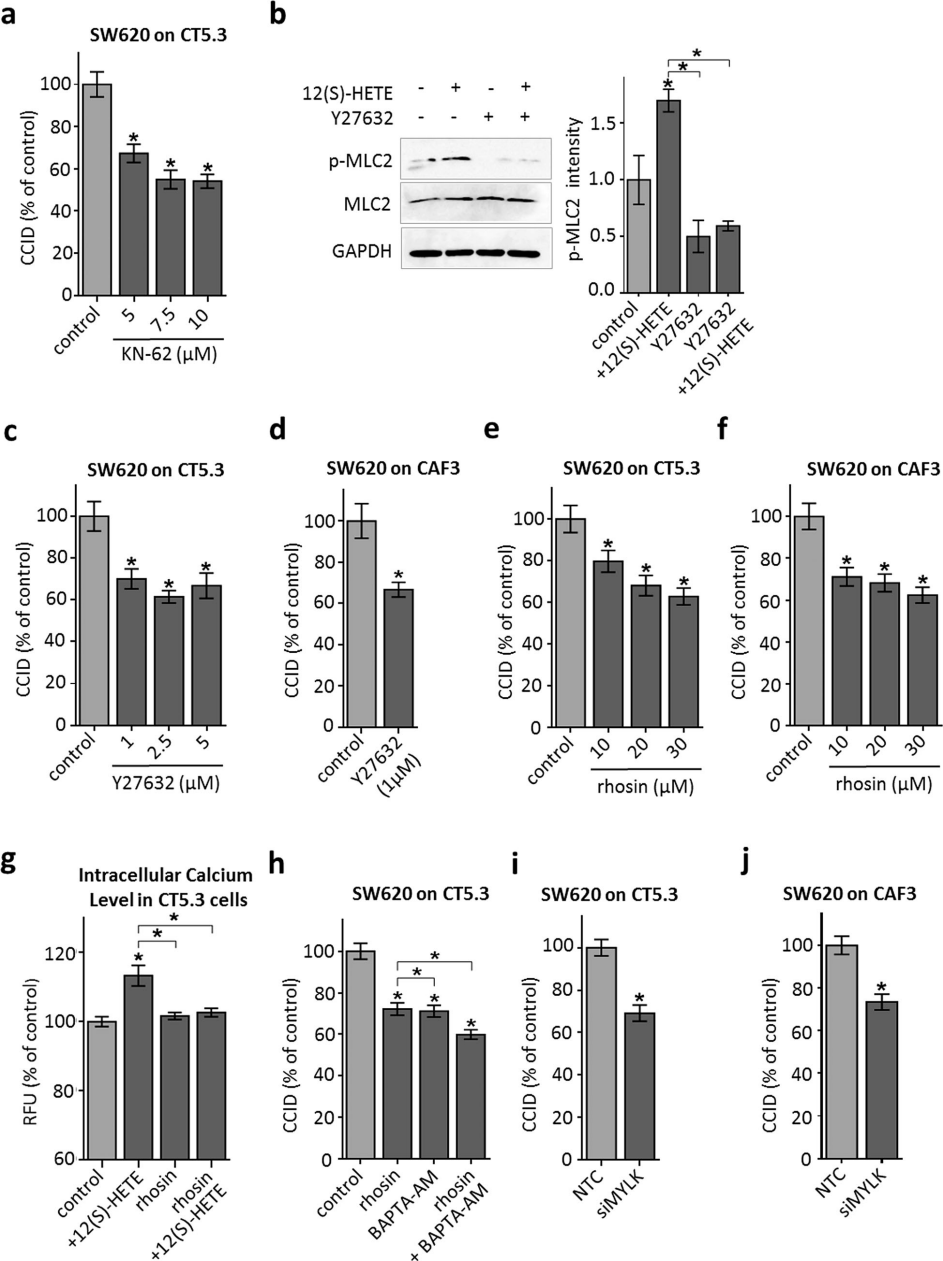
RHO/ROCK signalling is involved in CAF retraction. **a** CT5.3 were pre-treated with the CamK-II inhibitor KN-62 at indicated concentrations or solvent as control (control; DMSO). Subsequently, SW620 spheroids were placed on top of the fibroblasts for 6 h CCID areas were measured. **b** Cells were pre-treated with 2.5 µM of the ROCK inhibitor Y27632 or solvent and subsequently stimulated with 1 µM 12(S)-HETE or solvent (control; DMSO) for 15 min. Western blotting was used to determine MLC2 phosphorylation at serine 19. Equal sample loading was controlled by MLC2 total protein and GAPDH. Phospho-MLC2 (p-MLC2) was quantified by densitometry and normalised to MLC2 and GAPDH. Solvent treated control was set to 1. **c**–**f** CT5.3 and CAF3 were pre-treated with Y27632 and the RHO inhibitor rhosin at indicated concentrations or solvent as control (control; DMSO). Subsequently, SW620 spheroids were placed on top of the fibroblasts for 6 h CCID areas were measured. **g** CT5.3 were pre-treated with 20 µM rhosin or DMSO, stimulated with 1 µM 12(S)-HETE or solvent (control; DMSO) and intracellular-free calcium was measured after 3 min. **h** CT5.3 cells were pre-treated with 20 µM rhosin, 1 µM BAPTA-AM or both and solvent (DMSO) was used as control (control). Then, SW620 spheroids were placed on top of CT5.3 for 6 h and CCID areas were measured. **i**, **j** CT5.3 and CAF3 were transfected with either non-targeting RNA (NTC) or siRNA targeting MYLK (siMYLK). After 24 h SW620 spheroids were transferred on top of the CT5.3 monolayers and after 6 h co-cultivation CCID areas were measured. *Error bars* indicate mean ± SEM, *asterisks* significance compared to control or compared to the experimental points connected by brackets (*p* < 0.05; *t* test or ANOVA)

Next, we addressed how increased cellular Ca^2+^ levels may induce CAF retraction. Ca^2+^ activates Ca-calmodulin kinase II (CamK-II) and specific inhibition of CamK-II by treatment of CAFs with KN-62 significantly attenuated CCID formation (Fig. 4a) and MLC2 phosphorylation (Supplementary Fig. S4). In dendritic cells, CamK-II activates the RHO/ROCK axis, both are regulators of cellular mobility, thereby linking Ca^2+^ signalling to cell movement [29]. Here we demonstrate that this was also the case in CT5.3 fibroblasts, as treatment with the ROCK-inhibitor Y27632 attenuated 12(S)-HETE-induced MLC2 phosphorylation at Ser19 (Fig. 4b). Furthermore, CRC-triggered CCID formation was inhibited by Y27632 in CT5.3 (Fig. 4c) and CAF3 (Fig. 4d) and by the RHO inhibitor rhosin (Fig. 4e, f) in a dose-dependent manner, which inhibited MLC2 phosphorylation (Supplementary Fig. S4) as well. Therefore, the CamK-II/RHO/ROCK/MLC2 pathway transduces the 12(S)-HETE signal in CAFs, leading to retraction and opening the gate for tumour invasion in vitro.

Interestingly, 12(S)-HETE-induced increase in free intracellular Ca^2+^ levels was inhibited by rhosin (Fig. 4g), suggesting that RHO transduced signals not only downstream of CamK-II but also upstream of Ca^2+^ release thereby representing a kind of feedback loop. CamK-II was reported to activate RHO [30, 31] and RHO induces PLC-epsilon, Ca^2+^ release, and cytoskeleton modifications [32, 33]. Consistently, the inhibition of RHO together with the inhibition of Ca^2+^ release attenuated CCID formation additively (Fig. 4h). Another Ca^2+^-calmodulin kinase subtype, MYLK which activates MLC2 directly [34], contributed to CCID formation as well. This was demonstrated by siRNA-mediated depletion of MYLK in CT5.3 fibroblasts and CAF3, which attenuated CCID formation (Fig. 4i, j; proper knock-down of MYLK expression is shown in supplementary Fig. S5). Taken together, these data place Ca^2+^ as a central signal for retraction of CAFs triggered by CRC spheroids.

### FDA-approved pharmaceutical drugs inhibit 12(S)-HETE-induced Ca^2+^ release and CRC invasion in vitro

**Fig. 5:**
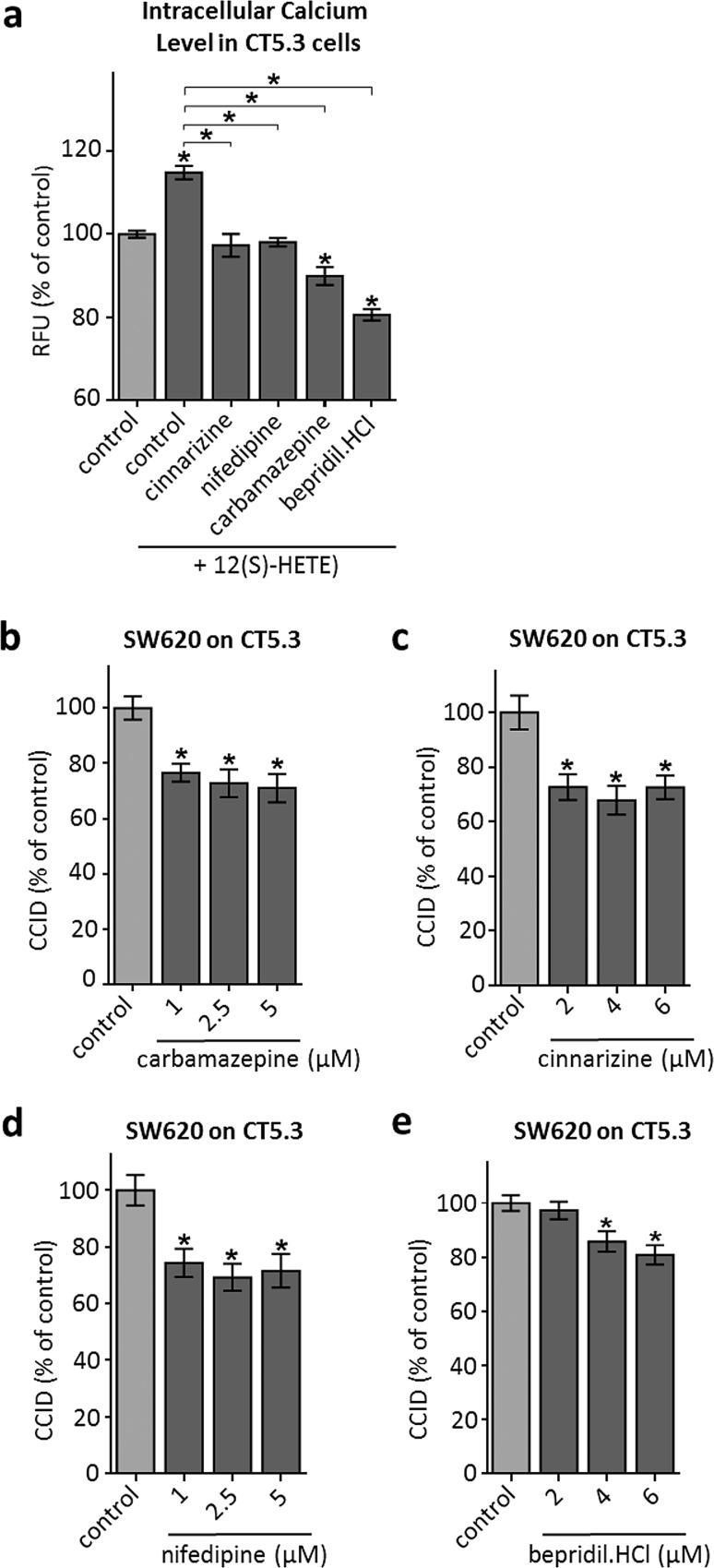
Inhibition of intracellular Ca^2+^ level increase and CCID formation by FDA-approved drugs. **a** CT5.3 were pre-treated with either carbamazepine, cinnarizine, nifedipine, bepridil hydrochloride or DMSO, stimulated with 1 µM 12(S)-HETE or solvent (control; DMSO) and intracellular-free calcium was measured after 3 min. **b**–**e** CT5.3 cells were pre-treated with either carbamazepine, cinnarizine, nifedipine and bepridil hydrochloride at indicated concentrations or solvent (control; DMSO). Then, SW620 spheroids were placed on top of the CT5.3 for 6 h and CCID areas were measured. *Bar graphs* represent means, *error bars* indicate ± SEM, *asterisks* significance compared to control (*p* < 0.05; *t* test or ANOVA)

To test the hypothesis that Ca^2+^ signalling is pivotal for CAF retraction, CT5.3 were pre-treated with FDA-approved drugs that reportedly influence Ca^2+^ availability by blocking respective ion channels. For this, we used carbamazepine (inhibiting L-type calcium channels), cinnarizine (inhibiting IP3-induced intracellular Ca^2+^ mobilisation), nifedipine (inhibiting L-type calcium channels specifically on vascular cells) and bepridil hydrochloride (inhibits binding to calmodulin only in the presence of calcium, thereby inhibiting MYLK and MLC2 phosphorylation) [35–38]. Each of these drugs inhibited Ca^2+^ release (Fig. 5a), as well as phosphorylation of MLC2 (except cinnarizine; Supplementary Fig. S4), and significantly attenuated SW620-mediated CCID formation in CT5.3 (Fig. 5b–e). Of note, in vitro retraction of CAFs was reduced at drug concentrations that can be principally achieved in humans.

## Discussion

Over time, malignant cancer cells acquire pro-invasive properties that are necessary to overcome a number of obstacles posed by the surrounding environment and, as an example, cancer cells reprogram their microenvironment to achieve metastatic outgrowth. In short, metastatic spreading involves remodelling of the basal matrix, local invasion through the adjacent stroma, transmigration into blood and lymph vessels, lymph node metastasis, mastering the shear stress within blood and lymph vessels, extravasation, and organ invasion. All these steps require a close cooperation of the tumour microenvironment. Therefore, understanding and manipulating of even just one of the many metastatic steps may provide a rationale for the prevention and the therapeutic management of metastasis. Yet, studies to delineate the contribution of stromal cells in this process mechanistically are scarce.

Honn and co-workers identified 12(S)-HETE as the “endothelial retraction factor” [6]. Neutrophils and monocytes are known to secrete 12(S)-HETE [5, 39] enabling them to transmigrate through endothelial cell (EC) barriers by triggering EC junction retraction. Likewise, MCF-7 breast cancer- [16, 40] and SW620 CRC cells [13] co-opt this mechanism and secrete 12(S)-HETE, which facilitates entering the lymphatic vasculature, travelling through the lymphatic endothelium and colonising lymph nodes. Baicalein is an accepted bona fide inhibitor of ALOX12/15, yet not entirely specific. Thus, the production of 12(S)-HETE by ALOX12/15 and its role for EC junction retraction and CCID formation was not only examined upon treatment with baicalein but also by an approach using shRNA against ALOX15 and the correlation between both inhibition experiments was direct and strict in a breast cancer/EC model [15]. As baicalein inhibited the retraction phenotype also in CRC/EC intravasation models [7, 15], it was also applied in the CRC/CAF model to highlight a role of ALOX12/15 in the invasion of CRC. CYP1A1 is another enzyme that produces 12(S)-HETE. CYP1A1 inhibition by siRNA and proadifen in breast cancer cells [10] or by proadifen in CRC cells [7] reduced the synthesis of 12(S)-HETE and the formation of CCIDs in EC monolayers as well and thus, further supports a role of 12(S)-HETE in the malignancy of CRC. Evidence for the pro-invasive contribution of 12(S)-HETE is provided by a CRC in vivo model in which the double amount of ALOX12-overexpressing SW480 cells [secreting four to fivefold more 12(S)-HETE as compared to control cells] [41] were detected in the vicinity of the lung blood vessels after transgrafting them into the rear flank of SCID mice [13]. ECs and CAFs are of mesenchymal origin and therefore quite mobile. 12(S)-HETE even increases their mesenchymal signature, which is reminiscent to “endothelial-to-mesenchymal-transition” (Endo-MT) in ECs [7, 14]. 12(S)-HETE also enhances the invasion of prostate cancer cells [42] potentially involving “epithelial-to-mesenchymal-transition” (EMT). Here, we elucidated the molecular/biochemical and cellular response of CAFs to CRC-derived 12(S)-HETE and demonstrate for the first time that 12(S)-HETE forced CAF retraction in vitro, which resulted in the formation of large gaps and upon contact with CRC spheroids both cell types dispersed into each other. It has to be underscored that the molecular adhesion between cancer cells and CAFs is an absolute prerequisite for CCID formation. However, the protagonists of inter-cellular adhesion are not yet identified in the here presented 3D model. Whether NF-kB and ICAM-1 establish CRC-CAF adhesion, as shown to be the case in 12(S)-HETE-triggered breast cancer/EC intravasation [14, 19], needs to be investigated in future studies.

We provide mechanistic evidence that the 12(S)-HETE-triggered signal in CAFs was mediated at least in part by the Ca^2+^/RHO/ROCK signalling axis ultimately leading to MLC2 activation, as all tested signal transduction inhibitors abrogated not only the 12(S)-HETE-induced signalling cascade and MLC2 phosphorylation (except cinnarizine), but also CCID formation. Most importantly, the knock-down of MLC2 inhibited CRC-triggered CCID formation in CAFs as well and this strongly argues for a contribution of 12(S)-HETE in the disintegration of the CAF barrier.

MLC2 expression and activation are the driving components of CAF mobility and cancer cell invasion. Also in ECs 12(S)-HETE activates MLC2, which significantly contributes to CCID formation [24, 43, 44]. Furthermore, it was shown that activated MLC2 in breast cancer cells accelerates their movement through the underlying endothelium [45]. Hence, during tumour progression, MLC2 seems to play a critical role in cancer cells and adjacent stromal cells. This is in accordance with the observation that tumour cell invasion is supported by the increased mobility of all involved cell types, which start to merge and mingle. However, the induction of MLC2 phosphorylation and CCID formation is not exclusively caused by the 12(S)-HETE gradient, as there are also other factors prompting tropism and retraction i.e. MMP1, which induces Ca^2+^ release and MLC2 activation in ECs as well [19, 46]. Yet, treating the CRC/CAF model with the pan-matrix metalloprotease inhibitor GM6001 did not inhibit the formation of CCIDs (data not shown). Therefore, this mechanism, which is known to contribute significantly in breast cancer/EC models, was not active during invasion of CRC into the CAF compartment.

Ca^2+^ availability and release are crucial steps in mediating retraction and mobility. Consistently, chelation of Ca^2+^ (by BAPTA-AM) together with simultaneous inhibition of RHO (by rhosin) impaired CCID formation within the CAF barrier, probably due to blocking the positive feed-back loop of RHO on Ca^2+^ release. Alternatively, the reduced CCID formation may have been due to simultaneous blocking of MYLK (another Ca^2+^-calmodulin kinase subtype directly activating MLC2) [34] together with the inhibition of RHO. MYLK is strongly expressed in CAFs as well as in IMR-90 normal lung fibroblasts (https://www.ebi.ac.uk/gxa/home) and we could demonstrate that primary normal human lung fibroblasts (HLF) respond in a similar way to SW620 spheroids as CAFs. Nearly as strong as MYLK is the expression of CamK-IId in IMR-90 fibroblasts, whereas the expression of CamK-IIg, CamK-I and CamK-III (EEF2K) is low (https://www.ebi.ac.uk/gxa/home; E-GEOD-26284; EMBL-EBI Expression Atlas, which contains gene expression data based on RNA sequencing). Hence, all of these CamKs may have contributed to the 12(S)-HETE-triggered and Ca^2+^-mediated fibroblast response that resulted in MLC2 phosphorylation and CCID formation. In line with the central role of elevated Ca^2+^ levels in response to 12(S)-HETE, we demonstrate that clinically used drugs, which were chosen on their property to affect calcium supply, inhibited the invasion of CRC spheroids through the CAF barrier. Of note, the here tested FDA-approved drugs diminished CAF retraction at concentrations that can be reached in humans. Therefore, it is tempting to speculate that interfering with Ca^2+^ availability might be a broadly applicable and promising route to combat metastasis in the clinic. This could be achieved in a short time frame, since such inhibitors are already approved and in use. However, one should have in mind that cancer cells may co-opt any mechanism (not just the one described herein) that has evolved to disintegrate, repulse and penetrate foreign tissue types.

## Electronic supplementary material

Below is the link to the electronic supplementary material.

Supplementary material 1 (TIFF 38121 kb)

Supplementary material 2 (TIFF 12724 kb)

Supplementary material 3 (TIFF 12724 kb)

Supplementary material 4 (TIFF 1456 kb)

Supplementary material 5 (TIFF 12725 kb)

## Abbreviations

ALOX12/15: Lipoxygenase 12/15
BAPTA-AM: 1,2-Bis(2-aminophenoxy)ethane-*N*,*N*,*N*’,*N*’-tetraacetic acid tetrakis(acetoxymethyl ester)
BEC: Blood endothelial cell
CamK-II: Ca^2+^-calmodulin kinase II
CRC: Colorectal cancer
CCID: Circular chemorepellent-induced defect
CAF: Cancer-associated fibroblast
EC: Endothelial cell
HLF: Human lung fibroblast
IP3: Inositol-3-phosphate
MLC2: Myosin light chain 2
MMP1: Matrix metallo-protease 1
MYLK: Myosin light chain kinase
PLC: Phospholipase C
RHO: Ras homologue gene family member
ROCK: Rho-associated protein kinase
siRNA: Small interfering RNA
3D: 3-Dimensional
12(S)-HETE: 12(S) hydroxyeicosatetraenoic acid
