# Supplementary figures and images for: Colon cancer cell-derived 12(S)-HETE induces the retraction of cancer-associated fibroblast via MLC2, RHO/ROCK and Ca2+ signalling
Source: Cell Mol Life Sci. 2016 Dec 24;74(10):1907–21. doi: 10.1007/s00018-016-2441-5 (PMC5390003; doi:10.1007/s00018-016-2441-5)

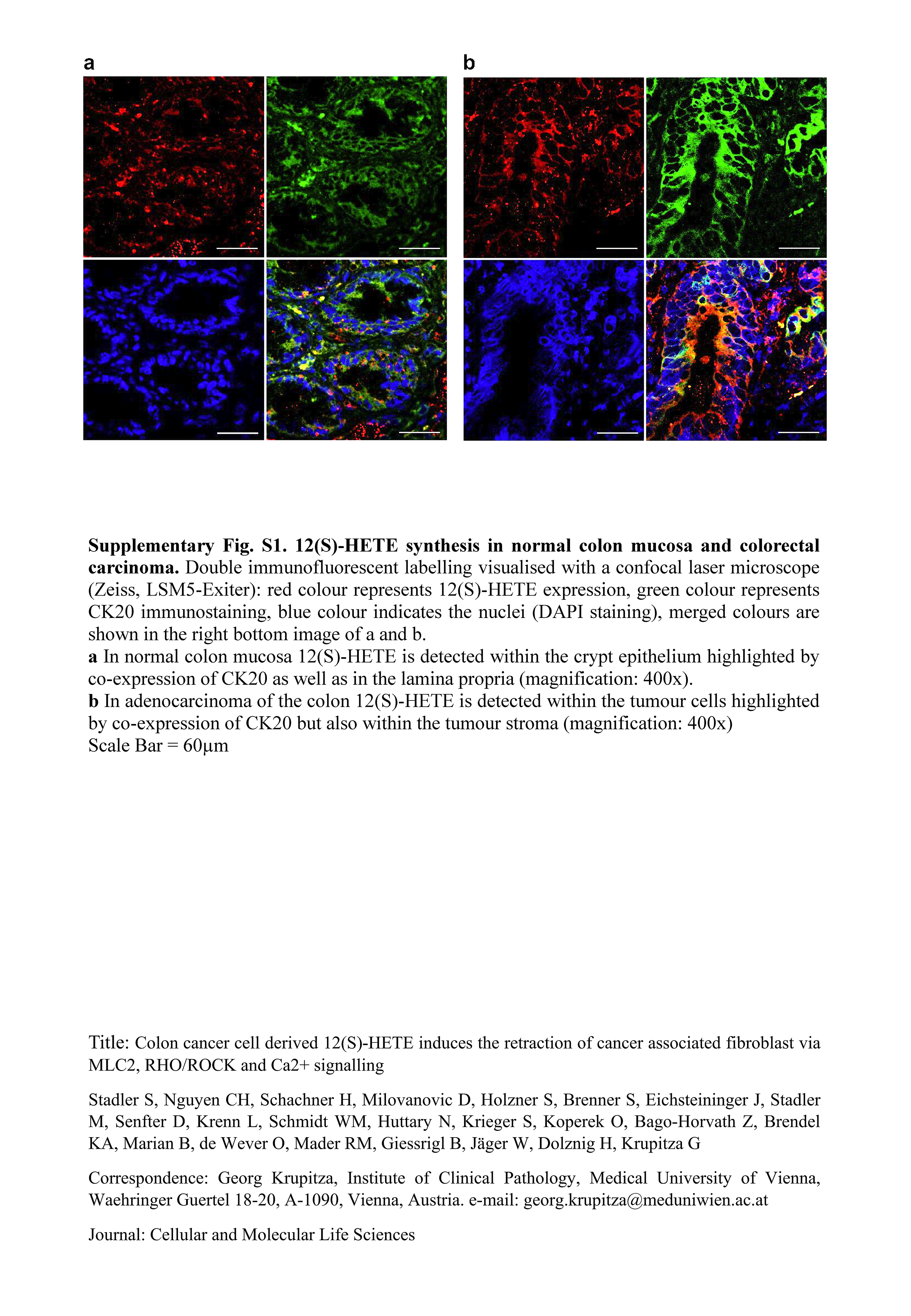

Supplement: Supplementary file 1 — Supplementary material 1 (TIFF 38121 kb) [file 18_2016_2441_MOESM1_ESM.tif]

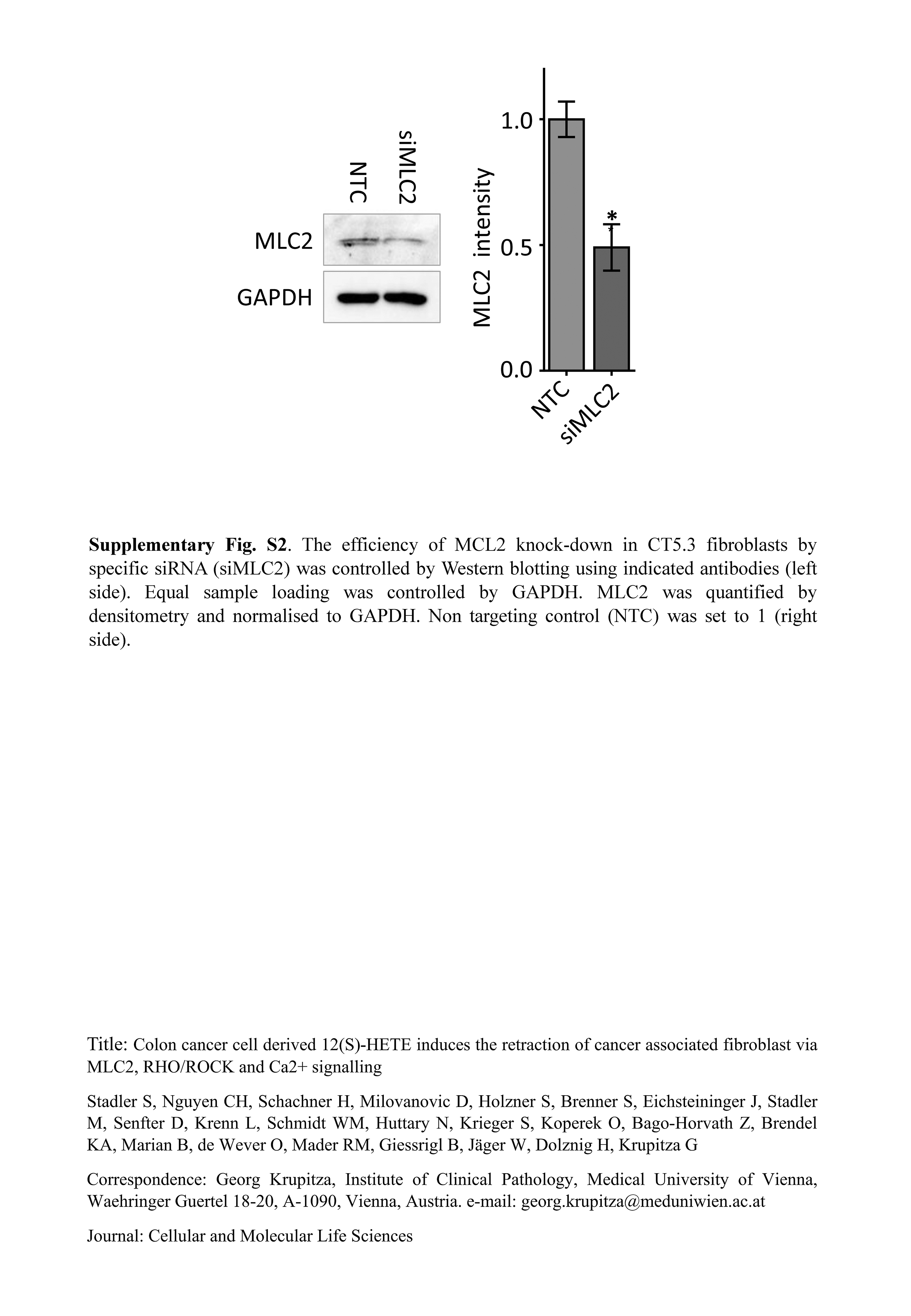

Supplement: Supplementary file 2 — Supplementary material 2 (TIFF 12724 kb) [file 18_2016_2441_MOESM2_ESM.tif]

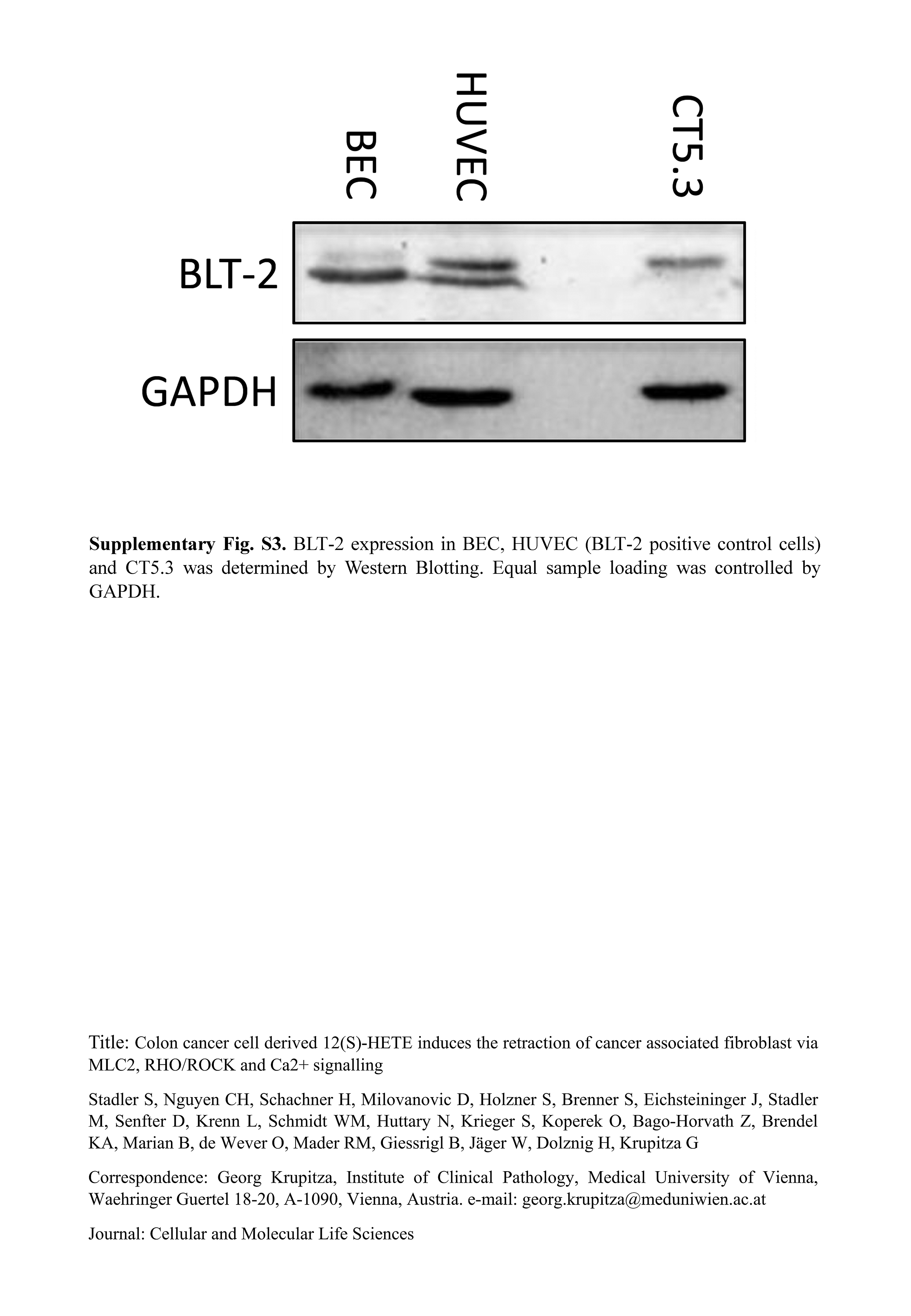

Supplement: Supplementary file 3 — Supplementary material 3 (TIFF 12724 kb) [file 18_2016_2441_MOESM3_ESM.tif]

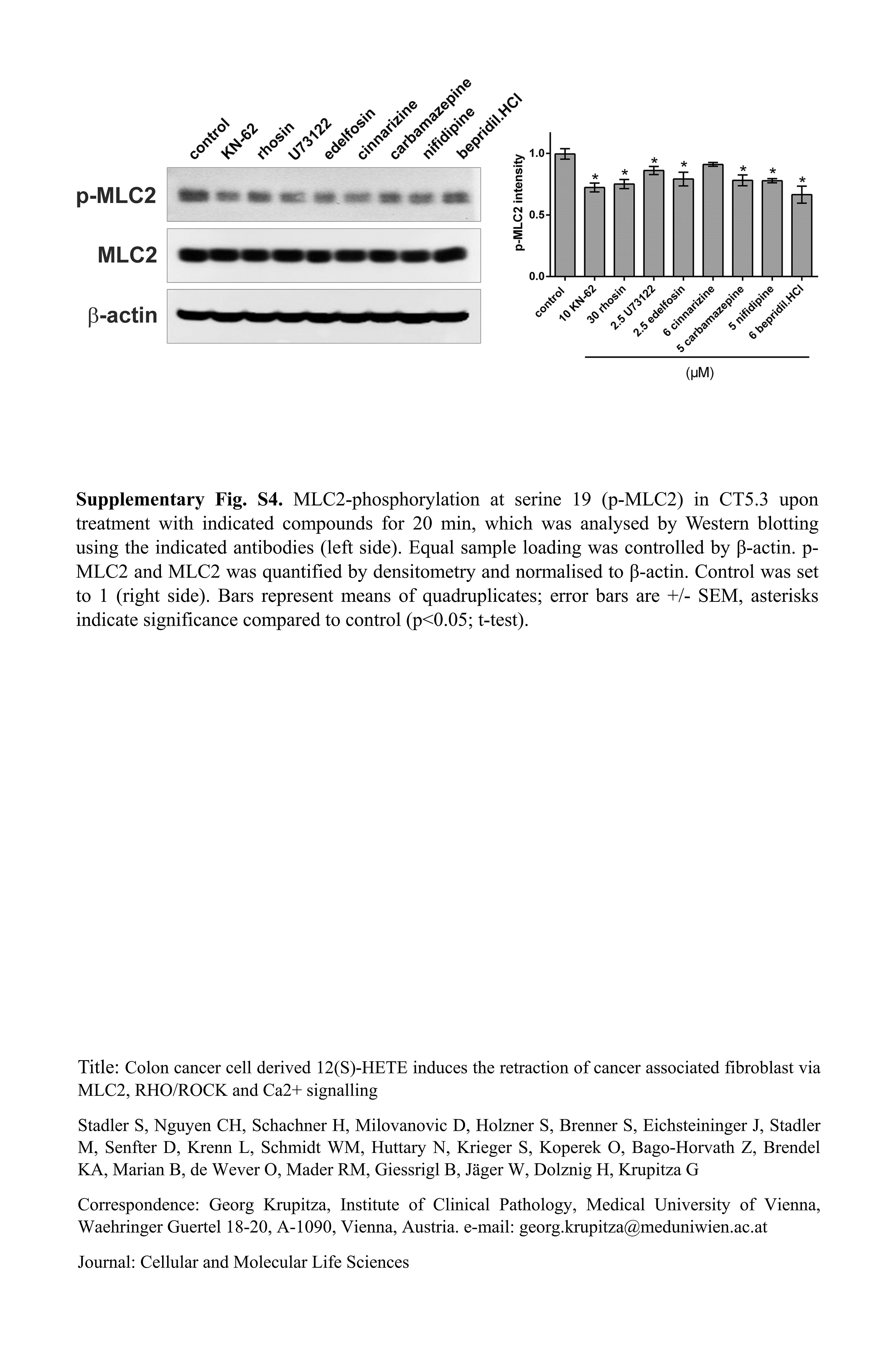

Supplement: Supplementary file 4 — Supplementary material 4 (TIFF 1456 kb) [file 18_2016_2441_MOESM4_ESM.tif]

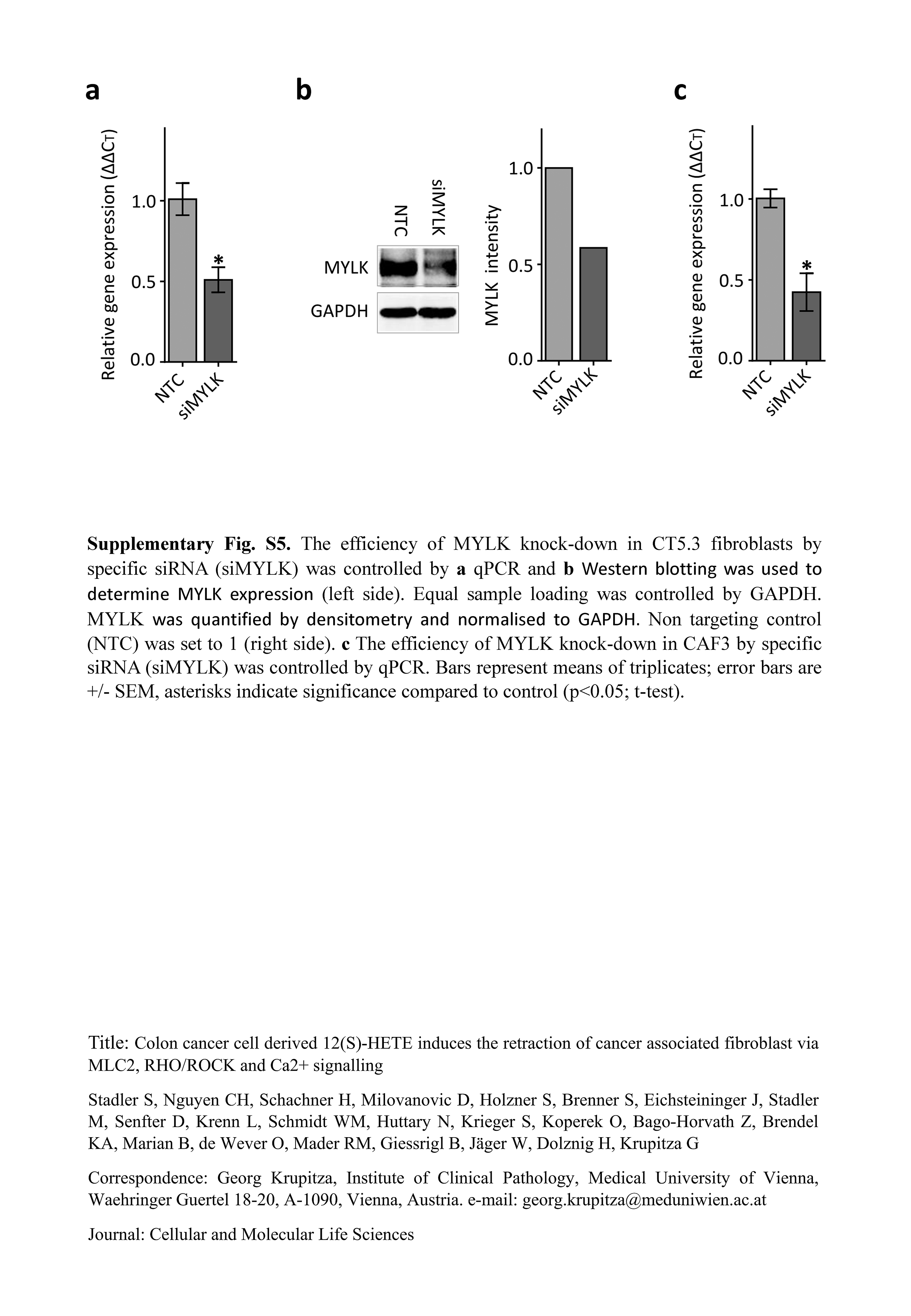

Supplement: Supplementary file 5 — Supplementary material 5 (TIFF 12725 kb) [file 18_2016_2441_MOESM5_ESM.tif]
